# *Zataria multiflora* and Pioglitazone Affect Systemic Inflammation and Oxidative Stress Induced by Inhaled Paraquat in Rats

**DOI:** 10.1155/2021/5575059

**Published:** 2021-05-04

**Authors:** Fatemeh Amin, Arghavan Memarzia, Ali Roohbakhsh, Farzaneh Shakeri, Mohammad Hossein Boskabady

**Affiliations:** ^1^Physiology-Pharmacology Research Center, Research Institute of Basic Medical Sciences, Rafsanjan University of Medical Sciences, Rafsanjan, Iran; ^2^Department of Physiology and Pharmacology, School of Medicine, Rafsanjan University of Medical Sciences, Rafsanjan, Iran; ^3^Applied Biomedical Research Center, Mashhad University of Medical Sciences, Mashhad, Iran 9177948564; ^4^Department of Physiology, School of Medicine, Mashhad University of Medical Sciences, Mashhad, Iran 9177948564; ^5^Pharmaceutical Research Center, Pharmaceutical Technology Institute, Mashhad University of Medical Sciences, Mashhad, Iran 9177948564; ^6^Natural Products and Medicinal Plants Research Center, North Khorasan University of Medical Sciences, Bojnurd, Iran; ^7^Department of Physiology and Pharmacology, School of Medicine, North Khorasan University of Medical Sciences, Bojnurd, Iran 7487794149

## Abstract

The effects of *Zataria multiflora* (*Z. multiflora*) and pioglitazone (a PPAR-*γ* agonist) alone and in combination, on systemic inflammation and oxidative stress induced by inhaled paraquat (PQ) as a herbicide, which induced inflammation in rats, were examined. Rats were exposed to (1) saline (control) and (2) 54 mg/m^3^ PQ aerosols (8 times, every other day, each time for 30 min) without treatment or treated with (3 and 4) two doses of *Z. multiflora* (200 and 800 mg/kg/day), (5 and 6) two doses of pioglitazone (5 and 10 mg/kg/day), (7) low doses of *Z*.*multiflora* + pioglitazone, (Pio-5+Z-200 mg/kg/day) or (8) dexamethasone (0.03 mg/kg/day) for 16 days, after the last PQ exposure. Different variables were measured at the end of the treatment period. Exposure to PQ significantly increased total and differential white blood cells (WBC) counts, serum levels of nitrite (NO_2_), malondialdehyde (MDA), interleukin- (IL) 17, and tumor necrosis factor alpha (TNF-*α*), but reduced thiol, superoxide dismutase (SOD), catalase (CAT), IL-10, and interferon-gamma (INF-*γ*) (*p* < 0.05 to *p* < 0.001). Most measured parameters were significantly improved in groups treated with either doses of the extract, pioglitazone, Pio-5+Z-200 mg/kg/day, or dexamethasone compared to the PQ group (*p* < 0.05 to *p* < 0.001). The combination of low doses of Pio-5+Z-200 mg/kg/day showed significantly higher effects compared to each one alone (*p* < 0.05 to *p* < 0.001). Systemic oxidative stress and inflammation due to inhaled PQ were improved by *Z. multiflora* and pioglitazone. Higher effects of Pio-5+Z-200 mg/kg/day compared to each one alone suggest modulation of PPAR-*γ* receptors by the plant extract, but further studies using PPAR-*γ* antagonists need to be done in this regard.

## 1. Introduction

Paraquat (PQ) (C12 H14 N2), a bipyridinium and nonselective quaternary nitrogen herbicide, is commonly used worldwide [1]. In 1985, only in Japan, approximately 2000 deaths occurred/year due to PQ digestion which were mostly intentional, and in 2020, more than 150,000 people died due to pesticide poisoning [2, 3]. PQ intoxication is characterized by swelling, bleeding, inflammation, and proliferation of bronchial epithelial cells [4]. Exposure to PQ is accidental or intentional in humans and animals, and the most common routes of exposure are inhalation and dermal [5]. Following oral administration, PQ causes burning and swelling of the mouth and throat, followed by gastrointestinal symptoms such as abdominal pain, loss of appetite, nausea, vomiting, diarrhea, and systemic inflammation [6]. It has also been reported that administration of PQ can lead to an increase in inflammatory factors such as TNF-*α* [7].

*Zataria multiflora* Boiss (*Z. multiflora*) from Lamiaceae family grows in southern Iran, Afghanistan, and Pakistan [8]. The constituents of *Z. multiflora* are terpenes, phenols, aliphatic alcohols, flavonoids, saponins, tannins, thymol, carvacrol, apigenin, luteolin, and 6-hydroxyluteolin glycosides, as well as di-, tri-, and tetramethoxylated. Oral administration of *Z. multiflora* aqueous extract (boiled) is used in traditional medicine for its analgesic, antiseptic, antioxidant, anti-inflammatory, anthelmintic, and antidiarrheal properties [8]. *Z. multiflora* extract has been used in inflammatory and immune deficiency diseases or against conditions associated with increased oxidative stress [8].

Peroxisome proliferator-activated receptors (PPARs) are a group of ligand-dependent nuclear receptors that act as transcription factors and have three known *α*, *β*/*δ*, and *γ* isoforms in humans [9]. PPAR-*γ* agonists were shown to affect the cardiovascular system [10]. PPAR-*γ* agonists are insulin-sensitive drugs used to treat insulin resistance [11]. The activation of PPAR-*γ* receptors showed anti-inflammatory and anticancer effects as well as the regulations of cellular metabolism, cell differentiation, and apoptosis [9, 12].

Therefore, in the present study, the effects of *Z. multiflora* hydroalcoholic extract and a PPAR-*γ* agonist and their combination, on systemic inflammation and oxidative stress induced by inhaled PQ in rats, were investigated. The effect of combination of low dose of the extract and pioglitazone was studied to evaluate their synergistic effect.

## 2. Materials and Methods

### 2.1. Animals and Groups

The study was performed in forty-eight male Wistar rats (weighing approximately 200–250 g) kept in the animal house, School of Medicine, Mashhad University of Medical Sciences, Iran. The animals were kept at 22 ± 2°C with a 12 h light/dark cycle and fed a standard diet and tap drinking water *ad libitum*. The ethics committee of Mashhad University of Medical Sciences approved the Animal Experiments of the present study with allowance Code 961202.

**Figure 1:**
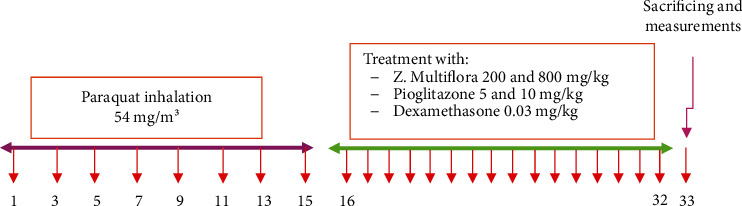
Protocol of exposing animals to t inhaled PQ (54 mg/m^3^) and treatment of animals with the extract, pioglitazone and dexamethasone.

Eight groups of rats (*n* = 6 in each group) were studied: (1) control group, which was exposed to normal saline aerosol; (2) animals exposed to PQ (Sigma-Aldrich Co., China) aerosol at dose of 54 mg/m^3^ [13–15]; (3 and 4) two groups exposed to PQ 54 mg/m^3^ and treated with two doses of the extract of *Z. multiflora* (200 and 800 mg/kg/day); (5 and 6) two groups exposed to PQ 54 mg/m^3^ and treated with two doses (5 and 10 mg/kg/day) of the pioglitazone (Samisaz Pharmaceutical Company, Iran); (7) one group exposed to PQ 54 mg/m^3^ and treated with Pio-5+Z-200 mg/kg/day; and (8) one group exposed to PQ 54 mg/m^3^ and treated with dexamethasone (Sigma-Aldrich Co., St. Louis, MO, Germany; 0.03 mg/kg/day). The control group was exposed to saline and other groups to PQ (Sigma-Aldrich Co., China) aerosols 8 times on days 1, 3, 5, 7, 9, 11, 13, and 15, each time for 30 min during a 16-day period. In treated groups, the extract, pioglitazone, or dexamethasone was administered by gavage for 16 days after the end of PQ exposure [15, 16] (Figure 1).

### 2.2. Exposure to PQ

For production of PQ aerosol, a nebulizer (Omron CX3, Japan, particle size 3–5 *μ*m) with an air flow of 8 L/min was used. A volume of 4.5 mL of 1.33 mg/mL PQ solution was added to the nebulizer chamber each time. The solution output of the nebulizer was 0.15 L/min and its air output was 3.7 L/min. The aerosol was delivered to exposure box, with dimensions 15 × 18 × 30 cm as previously described [15]. Therefore, the PQ dose in the exposure box was 54 mg/m^3^ [13].

### 2.3. Plant Extract Preparation

Plant collection and extract preparation were fully described in our previous study [15]. The plant was identified by Mr. Joharchi, Herbarium of the School of Agriculture, Ferdowsi University, and a voucher specimen was preserved (Herbarium No. 35314, FUMH). Briefly, the hydro-ethanolic extract was prepared by mixing 100 g of dried shoots and powdered *Z. multiflora* with 875 mL of 50% ethanol and shacked for 72 h at room temperature. The solvent was removed under reduced pressure, and the yield extract was 33.2 g. The studied doses of the extract were freshly prepared for gavage by adding water to dried extract [15].

### 2.4. Characterization of the Extract of Z. multiflora by HPLC

**Figure 2:**
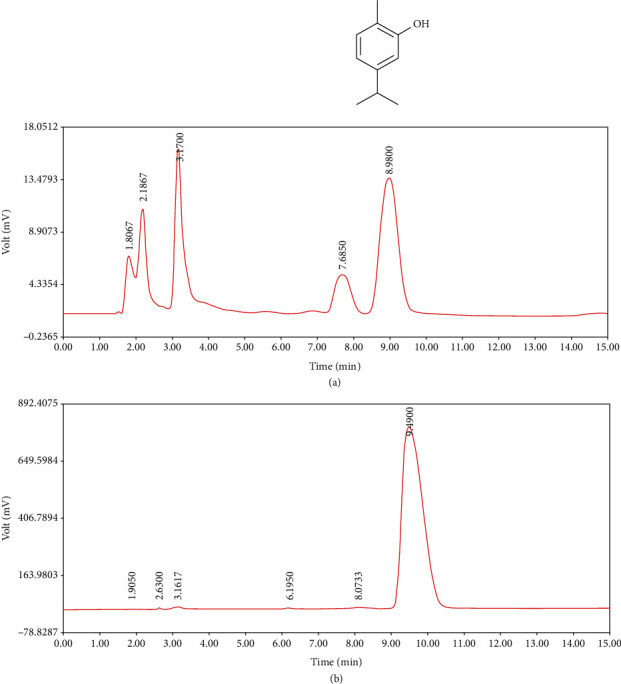
Fingerprint of (a) the extract of *Z. multiflora* (50 mg/mL) and the (b) pure carvacrol (C10H14O, 5/1000) (Boskabady et al., 2011). The extract showed a peak appears on 8.98 min which was as the same as retention time (RT) of pure carvacrol (9.49 min).

The extract of the plant was characterized in our previous study by a HPLC-UV (multiwavelengths) (Waters 474, Waters Corporation, Milford, MA, USA) finger print.  Figure 2 illustrates chromatographic profile of pure carvacrol (5/1000) with retention time at about 9 min.

### 2.5. Total and Differential WBC Counts

After the end of the treatment period (day 33), the rats were anesthetized by intraperitoneal injections of ketamine (50 mg/kg) and xylazine (5 mg/kg). Peripheral blood (2.5 mL) was prepared from the heart immediately after animal sacrificing. Then, 0.5 mL of blood was mixed with Turk solution, and total WBC number was determined in a hemocytometer (Burker chamber). For differential WBC count, the smear of blood was prepared and stained with Wright-Giemsa. Differential cell analysis was carried as previously described [17].

### 2.6. Oxidant and Antioxidant Biomarker Measurement

The blood samples (2 mL) were centrifuged at 2000 revolution per minute (rpm) for 10 minutes. Concentrations of oxidants biomarkers including malondialdehyde (MDA) and nitrite (NO_2_), as well as status of antioxidants including total thiol content, superoxide dismutase (SOD), and catalase (CAT) activities in the serum, were evaluated as previously described.

### 2.7. Cytokine Measurement

Serum levels of cytokines IL-10, IFN-*γ*, IL-17, and TNF-*α* were measured using specific enzyme-linked immunosorbent assay (ELISA) kits (Hangzhou Eastbiopharm, Iran) according to the manufacturer's protocol as previously reported.

### 2.8. Statistical Analysis

The normal distribution of the data was checked using the Kolmogorov-Smirnov test. Data were analyzed by one-way analysis of variance (ANOVA) followed by Tukey's multiple comparison test, and results are presented as the mean ± SEM. Values of *p* < 0.05 were considered statistically significant.

## 3. Results

### 3.1. Total and Differential WBC Counts

Total and differential WBC were increased in the blood of animals exposed to inhaled PQ compared to the control group (*p* < 0.05 for lymphocytes and *p* < 0.001 for other cases). Total WBC and neutrophil were reduced in all treated groups except for the group treated with low dose of the extract; eosinophil was reduced in groups treated with the two doses of pioglitazone, Pio-5+Z-200 mg/kg/day, and dexamethasone; lymphocyte was decreased in groups treated with high-dose pioglitazone, Pio-5+Z-200 mg/kg/day, and dexamethasone; and monocyte was reduced in groups treated with high-dose extract, Pio-5+Z-200 mg/kg/day, and dexamethasone (*p* < 0.05 to *p* < 0.001) (Table 1).

The effect of dexamethasone treatment on neutrophil was significantly higher than that of both doses of the extract, low-dose pioglitazone, and Pio-5+Z-200 mg/kg/day. Dexamethasone effect on monocyte was higher than both doses of the extract and pioglitazone, and its effect on eosinophil count was higher than both doses of the extract and low-dose pioglitazone; however, dexamethasone effect on lymphocyte was lower than the Pio-5+Z-200 mg/kg/day group (*p* < 0.05 to *p* < 0.001) (Table 1).

The effects of high-dose extract and pioglitazone treatment on total WBC and eosinophil and the effect of high-dose pioglitazone on neutrophil and lymphocyte were significantly higher than their low doses (*p* < 0.05 to *p* < 0.001) (Table 1).

In addition, treatment with low-dose pioglitazone + extract had significantly higher effects on total WBC and lymphocyte counts compared to low-dose extract and on monocyte than low-dose extract and pioglitazone alone (*p* < 0.001 for lymphocyte and *p* < 0.05 for other cases) (Table 1).

### 3.2. Oxidant and Antioxidant Biomarkers

**Figure 3:**
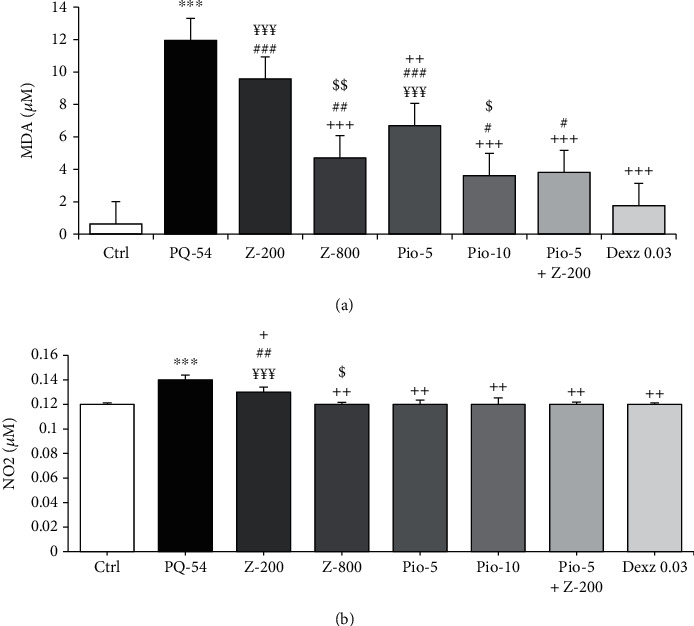
Serum levels of malondialdehyde (MDA) (a) and nitrite (NO_2_) (b) of control group (Ctrl), group exposed to paraquat aerosol at doses of 54 mg/m^3^ (PQ-54), and groups exposed to PQ-54 mg/m^3^ and treated with 5 and 10 mg/kg/day pioglitazone, 200 and 800 mg/kg/day *Zataria multiflora*, 0.03 mg/kg/day dexamethasone, and 5 mg/kg/day pioglitazone + 200 mg/kg/day*Zataria multiflora* (Pio-5, Pio-10, Z-200, Z-800, Dexa 0.03, and Pio‐5 + Z‐200, respectively). The results are expressed as the mean ± SEM (*n* = 6 in each group). ^∗∗∗^*p* < 0.001 compared to the control group. ^+^*p* < 0.05, ^++^*p* < 0.01, and ^+++^*p* < 0.001 compared to the PQ group; ^#^*p* < 0.05, ^##^*p* < 0.01, and ^###^*p* < 0.001 compared to treatment with other treated groups; ^$^*p* < 0.05 and ^$$^*p* < 0.01 compared to low dose of *Zataria multiflora* and pioglitazone groups; ^¥¥¥^*p* < 0.001 compared to the Pio‐5 mg/kg + Z‐200 mg/kg group. Comparisons between different groups were made using one-way ANOVA followed by Tukey's multiple comparison test.

**Figure 4:**
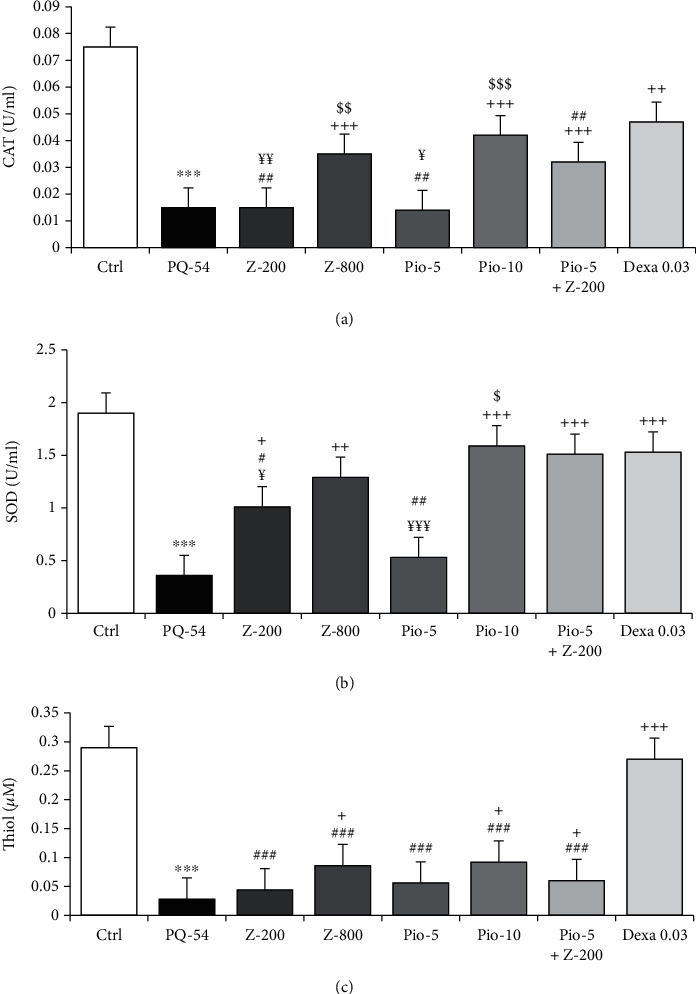
Serum levels of catalase (CAT) (a), superoxide dismutase (SOD) (b) activities, and thiol concentration (c) of the control group (Ctrl), group exposed to paraquat aerosol at doses of 54 mg/m^3^ (PQ-54), and groups exposed to PQ-54 mg/m^3^ and treated with 5 and 10 mg/kg/day pioglitazone, 200 and 800 mg/kg/day *Zataria multiflora*, 0.03 mg/kg/day dexamethasone, and 5 mg/kg/day pioglitazone + 200 mg/kg/day *Zataria* *multiflora* (Pio-5, Pio-10, Z-200, Z-800, Dexa 0.03, and Pio-5 + Z-200, respectively). The results are expressed as the mean ± SEM (*n* = 6 in each group). ^∗∗∗^*p* < 0.001 compared to the control group. ^+^*p* < 0.05, ^++^*p* < 0.01, and ^+++^*p* < 0.001 compared to the PQ group. ^#^*p* < 0.05, ^##^*p* < 0.01, and ^###^*p* < 0.001 compared treatment with other dexamethasone-treated groups. ^$^*p* < 0.05, ^$$^*p* < 0.01, and ^$$$^*p* < 0.001 compared to low dose of *Zataria multiflora* and pioglitazone groups. ^¥^*p* < 0.05, ^¥¥^*p* < 0.01, and ^¥¥¥^*p* < 0.001 compared to the Pio‐5 mg/kg + Z‐200 mg/kg group. Comparisons between different groups were made using one-way ANOVA followed by Tukey's multiple comparison test.

Significant increases in MDA and NO_2_ concentrations and significant decreases in total thiol content, and SOD and CAT activities were seen in the group exposed to inhaled PQ compared to the control group (*p* < 0.001 for all cases) (Figures 3 and 4).

The levels of NO_2_ in all treated groups, MDA level in all groups except low-dose extract, SOD activity except low-dose pioglitazone, and CAT and thiol levels in all treated groups except groups treated with low-dose extract and pioglitazone were significantly improved compared to the PQ group (*p* < 0.05 to *p* < 0.001) (Figures 3 and 4).

The effects of dexamethasone treatment on MDA and thiol levels were significantly higher than all other treated groups. Dexamethasone effect was significantly higher on CAT activity than all treated groups except for high-dose pioglitazone, on SOD activity than only treated groups with low-dose extract and pioglitazone and on NO_2_ level than only treated group with low-dose extract (*p* < 0.05 to *p* < 0.001) (Figures 3 and 4).

The effects of high-dose extract and pioglitazone treatment on MDA and CAT levels, the effect of high-dose extract on NO_2_ level, and the effect of high-dose pioglitazone on SOD activity were significantly higher than their low doses (*p* < 0.05 to *p* < 0.001) (Figures 3 and 4).

Treatment with Pio-5+Z-200 mg/kg/day had significantly higher effects on MDA, SOD, and CAT levels than low doses of the extract and pioglitazone and on NO_2_ level than low-dose extract alone (*p* < 0.01 for CAT and SOD and *p* < 0.001 for MDA and NO_2_) (Figures 3 and 4).

### 3.3. Serum Cytokine Level

**Figure 5:**
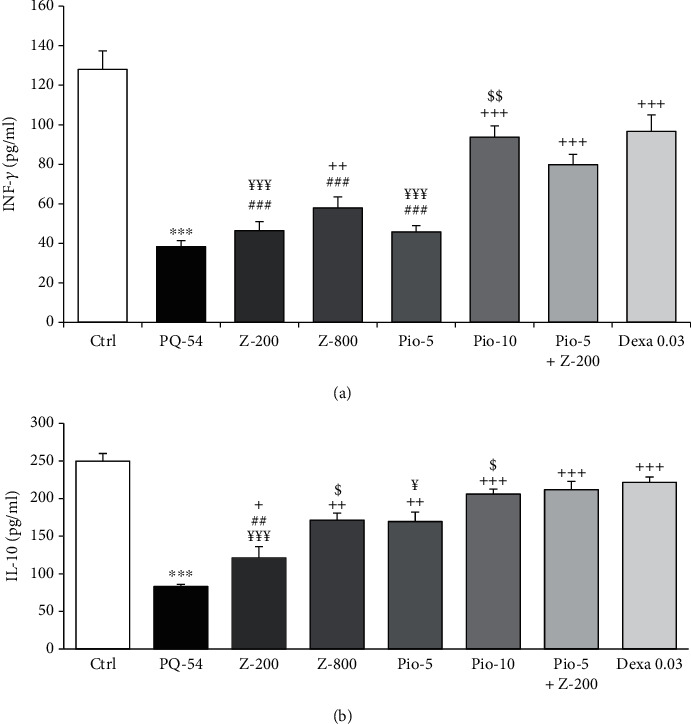
Values of interferon gama (INF-*γ*) (a) and interleukin-10 (IL-10) (b) in the serum of control group (Ctrl), group exposed to paraquat aerosol at doses of 54 mg/m^3^ (PQ-54), and groups exposed to PQ-54 mg/m^3^ and treated with 5 and 10 mg/kg/day pioglitazone, 200 and 800 mg/kg/day *Zataria multiflora*, 0.03 mg/kg/day dexamethasone, and 5 mg/kg/day pioglitazone + 200 mg/kg/day *Zataria* *multiflora* (Pio-5, Pio-10, Z-200, Z-800, Dexa 0.03, and Pio-5 + Z-200, respectively). The results are expressed as the mean ± SEM (*n* = 6 in each group). ^∗∗∗^*p* < 0.001 compared to the control group. ^+^*p* < 0.05, ^++^*p* < 0.01, and ^+++^*p* < 0.001 compared to the PQ group. ^##^*p* < 0.01 and ^###^*p* < 0.001 compared dexamethasone treatment with other treated groups. ^$^*p* < 0.05 and ^$$^*p* < 0.01 compared to low dose of *Zataria multiflora* and pioglitazone groups. ^¥¥¥^*p* < 0.001 comparison between Pio‐5 mg/kg + Z‐200 mg/kg with low dose of *Zataria multiflora* and pioglitazone groups. ^¥^*p* < 0.05 and ^¥¥¥^*p* < 0.001 compared to Pio‐5 mg/kg + Z‐200 mg/kg group. Comparisons between different groups were made using one-way ANOVA followed by Tukey's multiple comparison test.

**Figure 6:**
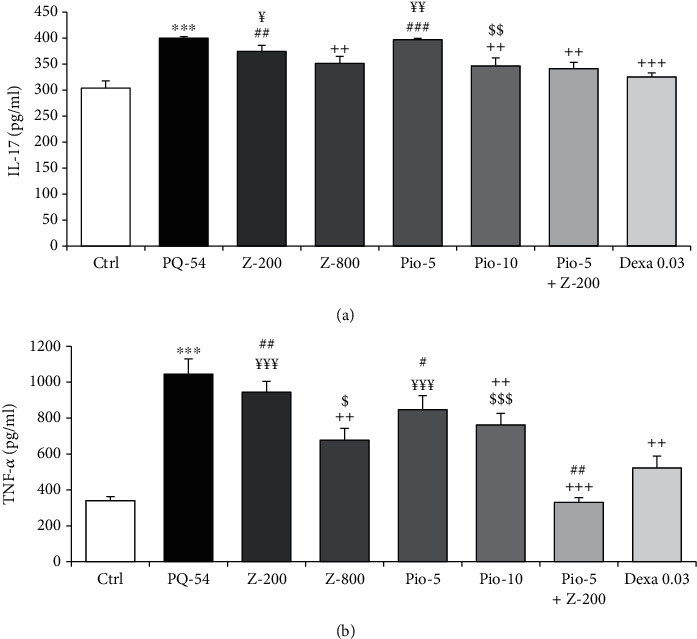
Values of IL-17 (a), INF-*α* (b) in serum of control group (Ctrl), group exposed to paraquat aerosol at doses of 54 mg/m^3^ (PQ-54), groups exposed to PQ-54 mg/m^3^ and treated with 5 and 10 mg/kg/day pioglitazone, 200 and 800 mg/kg/day *Zataria multiflora*, 0.03 mg/kg/day dexamethasone, and 5 mg/kg/day pioglitazone + 200 mg/kg/day *Zataria* *multiflora* (Pio-5, Pio-10, Z-200, Z-800, Dexa 0.03, and Pio‐5 + Z‐200, respectively). The results are expressed as the mean ± SEM (*n* = 6 in each group). ^∗∗∗^*p* < 0.001 compared to the control group. ^++^*p* < 0.01 and ^+++^*p* < 0.001 compared to the PQ group. ^#^*p* < 0.05, ^##^*p* < 0.01, and ^###^*p* < 0.001 compared dexamethasone treatment with other treated groups. ^$^*p* < 0.05, ^$$^*p* < 0.01, and ^$$$^*p* < 0.001 compared to low dose of *Zataria multiflora* and pioglitazone groups. ^¥^*p* < 0.05 and ^¥¥¥^*p* < 0.001 compared to Pio‐5 mg/kg + Z‐200 mg/kg group. Comparisons between different groups were made using one-way ANOVA followed by Tukey's multiple comparison test.

Serum levels of IL-17 and TNF-*α* were significantly increased, but IL-10 and INF-*γ* were decreased in PQ-exposed animals compared to the control group (*p* < 0.001 for all cases). Serum levels of IL-10 in all treated groups and INF-*γ*, TNF-*α*, and IL-17 in all treated groups except groups treated with low-dose extract and pioglitazone were significantly improved compared to the PQ group (*p* < 0.05 to *p* < 0.001) (Figures 5 and 6).

The effects of dexamethasone treatment were significantly higher on the IL-10 level compared to low-dose extract, on IL-17 and TNF-*α* than low-dose extract and pioglitazone, and on INF-*γ* than both doses of the extract and low-dose pioglitazone-treated groups (*p* < 0.01 and *p* < 0.001) (Figures 5 and 6). However, the effect of treatment with combination of Pio-5+Z-200 mg/kg/day was significantly higher on TNF-*α* than dexamethasone (*p* < 0.01) (Figure 6).

The effects of high-dose extract and pioglitazone treatment on IL-10 and TNF-*α* and the effect of high-dose pioglitazone on IL-17 and INF-*γ* levels were significantly higher than their low dose (*p* < 0.05 to *p* < 0.001) (Figures 5 and 6).

Treatment with Pio-5+Z-200 mg/kg/day was significantly higher effects on IL-10, IL-17, TNF-*α*, and INF-*γ* levels compared to low doses of the extract and pioglitazone alone (*p* < 0.05 to *p* < 0.001) (Figures 5 and 6).

## 4. Discussion

Total and all differential WBC were significantly increased due to inhaled PQ in the current study which are supported by the previous animal and human studies [18–21].

Treatment with *Z. multiflora* extract and pioglitazone decreased total and differential WBC counts in rats exposed to inhaled PQ in a concentration-dependent manner which was higher in the treated group with Pio-5+Z-200 mg/kg/day than low-dose pioglitazone or extract alone which showed a synergistic effect for these two agents. This synergistic effect may indicate the effect of Z*. multiflora* extract on PPAR-*γ* receptors. The activated PPAR-*γ* receptors and inhibited COX-2, by carvacrol, the main constituent of *Z. multiflora*, support the potential effect of *Z. multiflora* on PPAR-*γ* receptors [22].

Reductions of total and differential WBC in both the blood and the BALF in animal models of asthma and COPD [23] and in subjects exposed to sulfur mustard [8] were shown by the extract of *Z. multiflora* as well as by pioglitazone treatment in patients with metabolic syndrome [24, 25] which support the results of the current study.

The serum levels of NO_2_ and MDA were significantly increased, but SOD, CAT, and thiol were decreased in t PQ-exposed rats. Previous studies also showed reduction of SOD and CAT activity in animal models of PQ poisoning [26, 27], the role of free radical generation in PQ-induced injuries [28], and decreased SOD and CAT activities in animal lung tissues due to PQ administration [29]. A positive correlation between enhanced levels of oxidants and inflammatory mediators with administered PQ doses [30], increased oxidant, and decreased antioxidant markers in the hippocampus due to PQ poisoning were shown [6, 31], which support the findings of the present study.

Treatment with *Z. multiflora* extract and pioglitazone improved oxidative stress markers in an animal exposed to PQ which was supported by the previous studies indicating the effects of *Z. multiflora* extract on oxidative stress markers [8, 23, 32, 33]. Treatment with pioglitazone also reduced oxidant markers and increased antioxidants in animal exposed to inhaled PQ. Previous studies showed reduction of inflammation and oxidative stress by pioglitazone and rosiglitazone [34–36] which was consistent with the results of the present study. However, treatment of PQ-exposed animals by the combination of low-dose pioglitazone + *Z*.*multiflora* extract showed higher improvement effects on oxidant and antioxidant biomarkers compared to low-dose pioglitazone or *Z. multiflora* extract alone.

Increased serum levels of IL-17 and TNF-*α* and decreased levels of IL-10 and INF-*γ* were observed in PQ-exposed rats. Increased serum level of TNF-*α* in patients with acute PQ poisoning [37], increased IL-1 *β* and TNF-*α* nuclear factor kappa (NF-*κ*B) activity nuclear factor kappa (NF-*κ*B) activity, reduced IL-10 in the lung due to PQ administration in rats [35], increased inflammatory cytokines in PQ-poisoned individuals [37], decreased serum levels of anti-inflammatory cytokines [31], and enhanced gene expression for IL-4, TGF-*β*, IL-17, and TNF-*α* after PQ challenge were reported [38], supporting the results of this study.

Treatment of PQ-exposed animals with *Z. multiflora* extract and pioglitazone decreased serum levels of IL-17 and TNF-*α* but increased IL-10 and INF-*γ*. The immunomodulatory effects of *Z. multiflora* extract were comprehensively reviewed previously [31], and the effects of the plant on various cytokine levels in animal models of asthma and COPD [23], on gene expression of various cytokine in a mouse model of asthma [39], and on different cytokines in patients with lung disorders due to sulfur mustard exposure also [8] were shown supporting the results of the present study. Two doses of the extract were chosen according to our previous studies [12, 20, 21, 36] which showed its minimum and maximum effects. Pioglitazone treatment also affects intestinal TNF-*α* [40]; increased IL-4 but decreased IFN-*γ*, TNF-*α*, and IL-6 [41]; decreased lung adenoma [42]; decreased NO, TNF-*α*, IL-1*β*, IL-6, and IL-8; increased IL-4 and IL-10 levels in LPS-stimulated astrocytes [43]; and decreased degranulation and adhesion of neutrophils in LPS-induced lung injury [44]. The effects of *Z. multiflora* extract and pioglitazone on cytokine levels in PQ-exposed rats were supported by the above studies. The protective effect of carvacrol, one of the main constituents of this plant on lung inflammation induced by inhaled PQ, was also reported [45].

Higher effects of the combination of low-dose *Z*.*multiflora* extract + pioglitazone on serum cytokine levels compared to the effects of each one alone indicated a synergistic effect of the two agents suggesting the PPAR-*γ* receptor-mediated effect of *Z. multiflora* extract, which is supported by the effect of carvacrol on PPAR-*γ* receptors [22]. However, further studies examining the effect of *Z. multiflora* extract and PPAR-*γ* receptors antagonist are needed to confirm this suggestion.

Dexamethasone, a known anti-inflammatory used in this study as positive control drug, showed a similar effect as *Z. multiflora* extract and pioglitazone on measured variables in PQ-exposed rats. These results support the anti-inflammatory effects of *Z. multiflora* extract and pioglitazone and their combination on systemic inflammation induced by inhaled PQ.

In a previous preliminary study, the effects of the *Z. multiflora* and its constituents carvacrol on only MDA, NO2, IL-6, and IFN-*γ* and the ratio of the two cytokine [13] and, in another study, the effects of low dose of pioglitazone, the extract and combination of low dose pioglitazone and *Z multiflora*, and low dose of pioglitazone on CAT, NO2, and MDA as well as the serum levels of IL-6 and INF-*γ* and the ratio of the two cytokine on PQ-induced systemic inflammation were examined [46]. However, in the present study, the effect of two doses of *Z multiflora*, two doses of pioglitazone, and the combination of low dose of the extract and pioglitazone on systemic inflammation and oxidative stress induced by inhaled PQ was examined more precisely and in more variables in different animals. In fact, in the published paper, a definite conclusion regarding the synergistic effect of the extract and pioglitazone and the interaction of the extract on PPAR-*γ* receptors could not be suggested while in the present study this goal was achieved.

A preventive effect for *Z. multiflora* extract and pioglitazone, a PPAR-*γ* agonist on systemic inflammation and oxidative stress induced by inhaled PQ in rats similar to the effects of dexamethasone, was indicated. The synergistic effects of low-dose extract + pioglitazone also suggested that *Z. multiflora* effects could be mediated by PPAR-*γ* receptors, but this suggestion requires further investigations using PPAR-*γ* antagonists.

## Figures and Tables

**Table 1:** Total and differential WBC counts in the blood of control group (Ctrl), group exposed to paraquat aerosol at doses of 54 mg/m^3^ (PQ-54), groups exposed to PQ-54 mg/m^3^ and treated with 5 and 10 mg/kg/day pioglitazone, 200 and 800 mg/kg/day *Zataria multiflora*, 0.03 mg/kg/day dexamethasone, and 5 mg/kg/day pioglitazone + 200 mg/kg/day *Zataria* *multiflora* (Pio-5, Pio-1, Z-200, Z-800, Dexa 0.03, and Pio‐5 + Z‐200, respectively).

White blood cells (WBC)	Total WBC	Neutrophil	Lymphocyte	Monocyte	Eosinophil
Ctrl	4300 ± 620.48	1114.2 ± 138.6	3069.4 ± 410.17	50.4 ± 16.027	20.6 ± 3.9
PQ-54	12440 ± 679.4^∗∗∗^	7665.8 ± 585.22^∗∗∗^	4315.4 ± 595.14^∗∗∗^	388.6 ± 57.8^∗∗∗^	241.2 ± 57.06^∗∗∗^
Z-200	11438.3 ± 784.28^¥^	6533.91 ± 656.46^###^	3873.18 ± 192.06^¥¥¥^	235.71 ± 59^##,¥^	212.18 ± 23.49^###,¥¥¥^
Z-800	8303.3 ± 650^++,$^	5239.08 ± 679.16^++,###^	3055.66 ± 309.11	144.91 ± 19.96^++,#^	149.08 ± 17.96^++,$,###,¥¥^
Pio-5	9080 ± 492.3^++^	5430.4 ± 357.6^++,###^	3321.2 ± 243.2	265.4 ± 38.64^##,¥^	114.8 ± 14.82^++,##,¥¥^
Pio-10	7110 ± 1131.2^+++,$^	3219.5 ± 672.5^+++,$$$^	2580 ± 400.03^+,$^	201.6 ± 40.56^+,##^	25.6 ± 5.6^+++,$$$^
Pio-5 + Z-200	7946.66 ± 1415.4^+^	4614.76 ± 873.2^++,#^	2367.2 ± 464.4^++^	201.86 ± 35.66^++,#^	20.55 ± 9.43^+++^
Dexa 0.03	8860 ± 1449.3^+^	2323.4 ± 264.47^+++^	3826.2 ± 597.15	82.8 ± 38.88^+++^	33.2 ± 15.53^+++^

The results are expressed as the mean ± SEM (*n* = 6 in each group). ^∗∗∗^*p* < 0.01 compared to the control group. ^+^*p* < 0.05, ^++^*p* < 0.01, and ^+++^*p* < 0.001 compared to the PQ group; ^#^*p* < 0.05 and ^###^*p* < 0.001 compared to dexamethasone. ^$^*p* < 0.05 and ^$$$^*p* < 0.001 compared to low dose of *Zataria multiflora* and pioglitazone groups. ^¥^*p* < 0.05 compared to Pio‐5 mg/kg + Z‐200 mg/kg group. Comparisons between different groups were made using one-way ANOVA followed by Tukey's multiple comparison test.

## Data Availability

The data (excel format) used to support the findings of this study are available from the corresponding author upon request.
